# The clinical value of computed tomography Hounsfield unit for diagnosing palpable inguinal lymph node metastasis in patients with penile cancer

**DOI:** 10.3389/fonc.2025.1388390

**Published:** 2025-02-10

**Authors:** Yu Li, Yu Chen, Gansheng Xie, Gang Li, Huming Yin

**Affiliations:** ^1^ Department of Urology, The First Affiliated Hospital of Soochow University, Suzhou, Jiangsu, China; ^2^ Department of Geriatrics, The First Affiliated Hospital of Soochow University, Suzhou, Jiangsu, China

**Keywords:** penile cancer, computed tomography, Hounsfield unit, inguinal lymph node, metastasis

## Abstract

**Background:**

Computed tomography (CT) Hounsfield units (HUs) of pathologically confirmed metastatic inguinal lymph nodes (ILNs) were proved to be higher than negative ones. We designed this study to explore the clinical value of CT HU for diagnosing palpable ILN metastasis in patients with penile cancer.

**Methods:**

A total of 32 patients with penile cancer, including 84 palpable ILNs, were recruited in this study. They all performed 5-mm layer pelvic contrast-enhanced CT (CE-CT) before treatment. The palpable ILNs were matched with CT image. By using radiologic software PACS, the layer with a maximum cross-sectional area of target lymph node was selected, and the short axis was defined as diameter. We outlined the edge of target lymph nodes, and the software automatically calculated its area, maximum CT HU, and average CT HU. All target ILNs were biopsied by surgery to confirm the presence of metastasis.

**Results:**

Compared with non-metastatic ILNs, metastatic ILNs had larger diameter, area, maximum non-contrast CT (NC-CT) HU, maximum arterial-phase CE-CT (ACE-CT) HU, average NC-CT HU, and average ACE-CT HU, with statistically significant differences (P < 0.05). Receiver operating characteristic analysis showed the all six parameters (maximum NC-CT HU, maximum ACE-CT HU, average NC-CT HU, average ACE-CT HU, diameter, and area) had significant diagnostic value for ILN metastasis, with an area under the curve of 0.847, 0.853, 0.900, 0.919, 0.809, and 0.789, respectively. The average ACE-CT HU (cutoff: 40.5) had the highest accuracy as 0.857, and maximum NC-CT HU (cutoff: 51.5) had the highest sensitivity of 0.897.

**Conclusion:**

ILN CT HU was clinically valuable for the diagnosis of palpable ILN metastasis in patients with newly diagnosed penile cancer.

## 1 Introduction

Penile cancer was once a common male carcinoma before the 1950s in China. As the basic health conditions improved and the popularity of circumcision increased, penile cancer has become a rare disease nowadays. The overall incidence reported in 2011 was 0.6/10^5^ (1).

The pathology of 95% penile cancers belongs to squamous cell carcinoma (2), and some patients have inguinal lymph node (ILN) metastasis on the time of diagnosis. Positive ILN is significantly associated with prognosis: the 5-year survival rate is 95%–100% in patients without ILN metastasis, and it drops to 50%–80% in the presence of ILN metastasis (3). Twenty percent of patients with penile cancer have palpable lymph nodes in the groin area at the time of presentation (4). However, only 70% of palpable lymph nodes are metastatic, and the rest are due to ulceration or inflammation of the penis (5). Conventionally, 4 to 6 weeks of anti-inflammatory treatment is recommended when ILNs can be palpated. If the lymph nodes do not shrink, bilateral ILN resection is suggested. However, this method has some shortcomings. First, observational waiting for months may delay the treatment and cause distant lymph node metastasis. Second, it is reported that 20% of non-palpable lymph nodes can be metastatic (6). Therefore, some authors suggest that an early lymphadenectomy is necessary for intermediate and high risk patients, with or without palpable ILNs (7, 8). In patients with suspicious ILN, ultrasound-guided fine-needle aspiration cytology can be an option (9), but this method needs special devices and is traumatic for patients. In clinical practice, we find that the computed tomography (CT) Hounsfield units (HUs) of pathologically confirmed metastatic ILNs are higher than negative ones. It is speculated that CT HU may have significant correlation with ILN metastasis. Therefore, we designed this prospective clinical study to research the diagnostic value of CT HU in penile cancer.

## 2 Methods

### 2.1 Patients

From January 2020 to August 2024, 45 patients with penile cancer were treated in the Urology department of our hospital. Thirty-two of them with palpable ILNs at diagnosis were enrolled in this study. No patients had histories of previous cancers or surgeries on the pelvic region. All the 32 patients received 5-mm layer pelvic contrast-enhanced CT (CE-CT) before surgery. A penile tumor resection (partial penectomy) plus palpable ILN biopsy was performed. Frozen section pathology of biopsied lymph nodes was implemented immediately. If the biopsy was confirmed metastatic (**Supplementary Figure S1**), then further bilateral inguinal lymphadenectomy was performed.

### 2.2 CT scanning technique

Patients were examined in a supine position using a CT scanner (Philips 256iCT, Amsterdam, Denmark). Scanning parameters included 120 kVp, 200–350 mAs, 1.0 helical pitch, and 5-mm-thick reconstructed sections. First, a pelvic non-contrast CT (NC-CT) scan was performed. Then, 100 mL of iohexol (Omnipaque, GE China, concentration of 350 mg of I/mL) was injected intravenously at a speed of 4 mL/s. Seven seconds after the CT HU of the descending aorta at the level of celiac trunk reaching 150, an arterial-phase CE-CT (ACE-CT) was launched. The average ACE-CT scanning time was 5 s.

### 2.3 Localization of palpable lymph nodes on pelvic CT

Before the biopsy, we located the palpable ILN on CT image, according to the size of ILN and the adjacent relationship between lymph nodes and the fixed anatomy (such as inguinal ligament, femoral artery, and spermatic cord).

### 2.4 Measurement of biopsy lymph nodes on CT image

**Figure 1:**
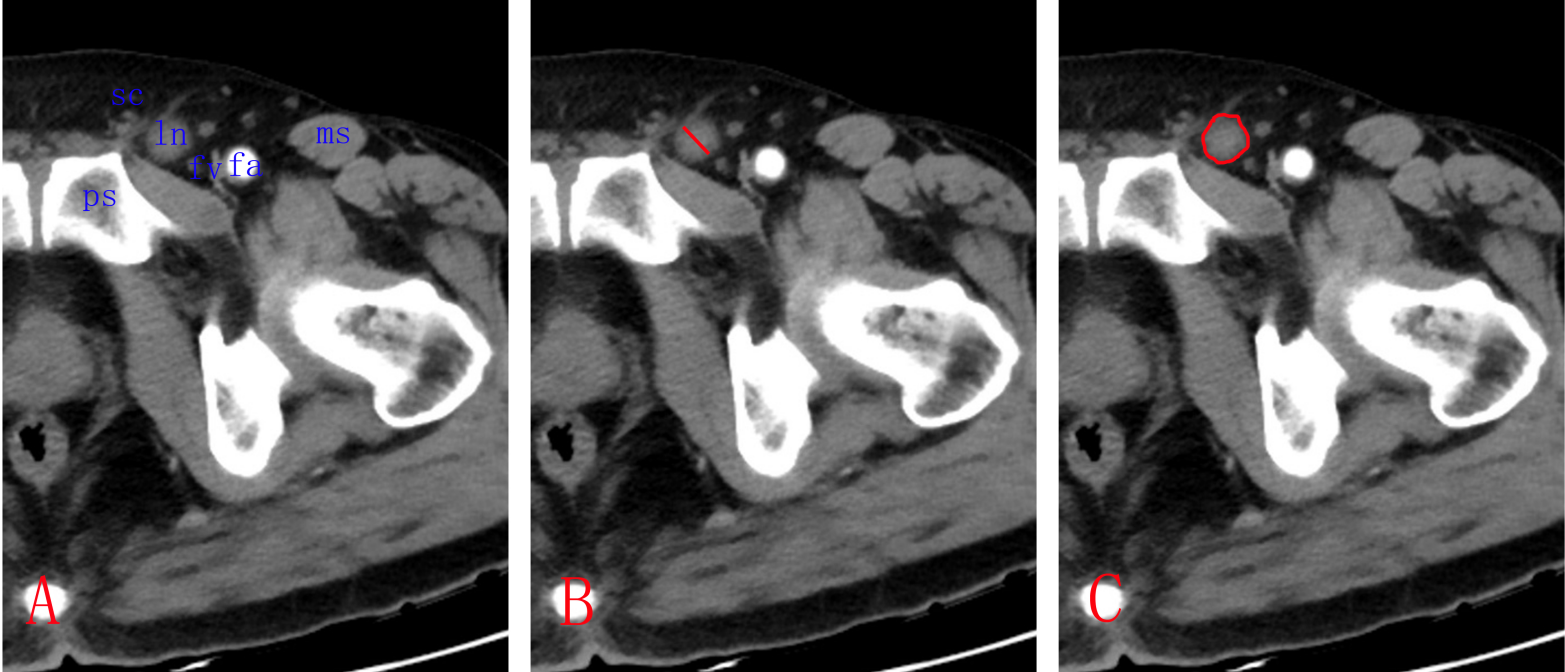
Measurement of inguinal lymph node (ILN) in patients with penile cancer. **(A)** Choosing the maximum sectioned layer of the target lymph node on the CT image with 5-mm slices. **(B)** Measuring the short axis diameter of ILN. **(C)** Tracing the outline of ILN by hand to include the whole lymph node. ln, lymph node; sc, spermatic cord; fv, femoral vein; fa, femoral artery; ps, pubic symphysis; ms, muscle sartorius.

We analyzed the NC-CT and ACE-CT images on the reading software PACS version 5.5 (Picture Archiving and Communication software, Neusoft, China). The layer with maximum cross-sectional area of a target lymph node was selected. The short axis across the lymph node was defined as diameter. To measure lymph node CT HU, we outlined the edge of lymph nodes by hand. As ILNs are surrounded by fat tissue, with great difference of CT density between the two structures, the edges of target lymph nodes are clear and sharp (**Figure 1**). The software automatically calculated the area, maximum CT HU, and average CT HU of target lymph nodes.

### 2.5 Pathological staging and grading of penile cancer

According to American Joint Committee on Cancer (AJCC) Cancer Staging Manual of penile cancer (eighth edition, New York, Springer International Publishing, 2017), the pathological T stage and N stage were determined for each patient. The primary tumor was graded as low differentiation, moderate differentiation, or high differentiation by pathological results.

### 2.6 Statistical analyses

Data were evaluated using SPSS version 17.0 software (IBM SPSS, Chicago, IL). Data were presented as mean ± standard deviation. Student’s *t*-test was used to assess differences between two groups. Chi-square test was used to compare constituent ratios of different groups. To explore the value of each parameter for ILN metastasis diagnosis, a receiver operating characteristic (ROC) curve was plotted and the resulting area under the curve (AUC) was calculated. Youden index [calculated as (sensitivity + specificity) − 1] was used to determine cutoff values. Differences were considered to be significantly difference if *P* < 0.05 (bilateral).

## 3 Results

We included 32 patients with a total of 84 ILNs biopsied. The average number of ILNs biopsied in one patient was 2.6. Thirteen patients were diagnosed as pT1 stage, and the other 19 patients were diagnosed as pT2 stage. There was no pT3 or pT4 in the study. Thirty-nine metastatic ILNs (46.4%) were diagnosed in 20 patients (62.5%). Their descriptive parameters are shown in **Table 1**.

**Table 1:** Clinical characteristics of the 32 patients.

Parameters		
Age (years)	58.9 ± 11.7 (29–79)
No. of ILNs biopsied per patient	2.6 1.5 (1–6)
Pathological T stage (total patients/patients with ILN metastasis)	
T1	13/8
T2	19/12
Primary tumor grading (total patients/patients with ILN metastasis)	
High differentiation	10/2
Moderate differentiation	10/7
Low differentiation	12/11
Lymph node metastasis	
Positive	39
Negative	45

Data are presented as m ± SD (range), n, n/n.

ILN, inguinal lymph node.

### 3.1 Association between ILN metastasis and clinical characteristics

No statistical difference in age was observed between metastatic patients and non-metastatic ones (60.5 ± 14.2 vs. 59.2 ± 11.8, *t* = 0.299, *P* = 0.767). The proportions of metastatic patients in pT1 and pT2 stage were 61.5% (8/13) and 63.2% (12/19), respectively, with no statistically significant difference (χ^2^ = 3.250, *P* = 0.355). However, the proportion of metastatic patients with high differentiation, moderate differentiation, and low differentiation penile cancers was 20% (2/10), 70% (7/10), and 83.3% (11/12), respectively, with statistically significant association between ILN metastatic status and tumor differentiation (χ^2^ = 9.751, *P* = 0.008). The results showed that patients with poorly differentiated cancers were more likely to develop ILN metastasis.

### 3.2 Comparative analysis of CT HU, diameter, and area for ILN metastatic status

Compared with non-metastatic ILNs, metastatic ILNs had larger maximum NC-CT HU, maximum ACE-CT HU, average NC-CT HU, average ACE-CT HU, diameter, and area. All the six parameters were statistically different between the two groups (P < 0.05). The results are presented in **Table 2**.

**Table 2:** Comparison of CT HU, diameter, and area between metastatic ILNs and non-metastatic ILNs.

Parameters	Metastatic ILNs	Non-metastatic ILNs	Statistics	*P*
n	39	45		
Average NC-CT HU	29.7 ± 15.2	1.19 ± 22.3	7.463	<0.001
Average ACE-CT HU	53.1 ± 19.8	13.3 ± 25.8	7.981	<0.001
Maximum NC-CT HU	63.1 ± 12.8	41.3 ± 17.3	6.604	<0.001
Maximum ACE-CT HU	96.3 ± 24.8	62.9 ± 21.8	6.562	<0.001
Diameter (mm)	19.6 ± 10.6	11.0 ± 2.8	4.891	<0.001
Area of ILN (mm^2^)	294.2 ± 368.1	75.8 ± 41.2	3.685	0.001

Data are presented as n, m ± SD.

NC-CT, non-contrast computed tomography; ACE-CT, arterial-phase contrast-enhanced computed tomography; HU, Hounsfield unit; ILN, inguinal lymph node.

### 3.3 Diagnostic value of CT HU, diameter, and area on ILN metastasis

**Figure 2:**
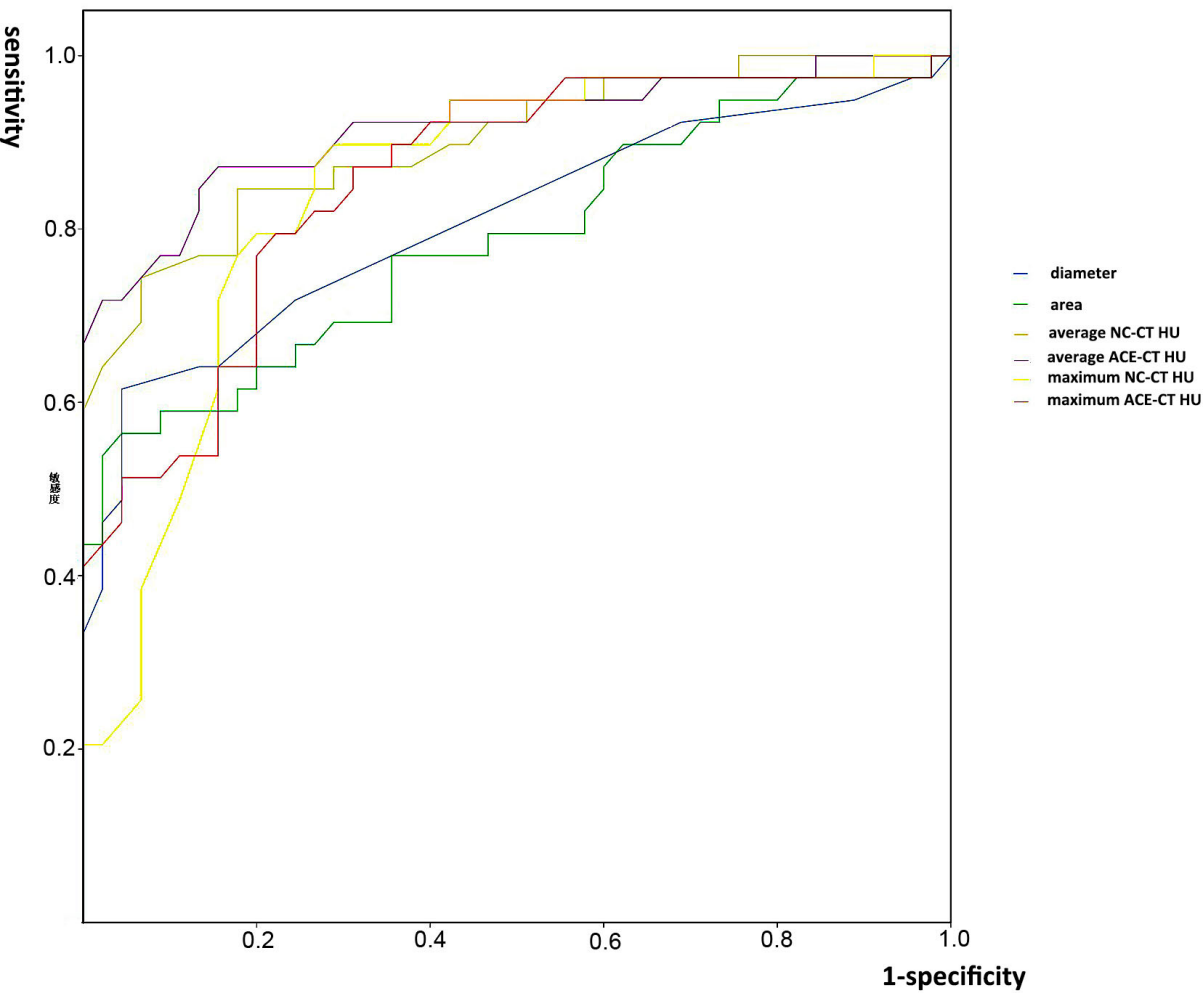
The ROC curves and areas under the curve (AUC) of the six parameters. NC-CT, non-contrast computed tomography; ACE-CT, arterial-phase contrast-enhanced computed tomography; HU, Hounsfield unit; ILN, inguinal lymph node.

The results of ROC curve were shown in **Figure 2**. Six parameters (maximum NC-CT HU, maximum ACE-CT HU, average NC-CT HU, average ACE-CT HU, diameter, and area) were analyzed. All of them were proved to have significant diagnostic value, and an AUC of each parameter is shown in **Table 3**. The average ACE-CT HU had the largest AUC as 0.919. The sensitivity, specificity, positive predictive value, negative predictive value, and accuracy of each parameter for the diagnosis of metastatic ILN are shown in **Table 4**. Among them, the average ACE-CT HU (cutoff: 40.5) had the highest accuracy as 0.857; maximum NC-CT HU (cutoff: 51.5) had the highest sensitivity of 0.897; both area and diameter had the highest specificity as 0.956.

**Table 3:** Areas under the curve (AUC) of six parameters.

Parameters	AUC	95% CI	*P*
Average NC-CT HU	0.900	0.834–0.967	<0.001
Average ACE-CT HU	0.919	0.856–0.981	<0.001
Maximum NC-CT HU	0.847	0.762–0.932	<0.001
Maximum ACE-CT HU	0.853	0.772–0.935	<0.001
Diameter (mm)	0.809	0.711–0.906	<0.001
Area of ILN (mm^2^)	0.789	0.688–0.889	0.001

NC-CT, non-contrast computed tomography; ACE-CT, arterial-phase contrast-enhanced computed tomography; HU, Hounsfield unit; ILN, inguinal lymph node; CI, confidence interval.

**Table 4:** The diagnostic value of six parameters on ILN metastasis.

	Cutoff	Sensitivity	Specificity	PPV	NPV	Accuracy
Average NC-CT HU	21.0	0.846	0.822	0.805	0.860	0.833
Average ACE-CT HU	40.5	0.872	0.844	0.829	0.884	0.857
Maximum NC-CT HU	51.5	0.897	0.711	0.729	0.889	0.798
Maximum ACE-CT HU	81.5	0.795	0.778	0.756	0.814	0.786
Diameter (mm)	15.5	0.615	0.956	0.923	0.741	0.809
Area of ILN (mm^2^)	150.0	0.564	0.956	0.917	0.717	0.798

NC-CT, non-contrast computed tomography; ACE-CT, arterial-phase contrast-enhanced computed tomography; HU, Hounsfield unit; ILN, inguinal lymph node; NPV, negative predictive value; PPV, positive predictive value.

## 4 Discussion

Palpable ILN in patients with penile cancer should be suspicious as metastasis. However, some of them in patients with newly diagnosed penile cancer are non-metastatic, caused by inflammatory reaction. In this study, only 39 of the 84 palpable lymph nodes (46.4%) had metastases. Physical examination can check the hardness, mobility, and adhesion of ILN and is vital to evaluate the N stage of penile cancer before treatment (10). Ultrasonography is a convenient method to detect enlarged ILN. Fine-needle aspiration cytology guiding by ultrasonography was reported to accurately stage patients with both impalpable and palpable ILN, with the sensitivity and specificity values of 87.3% and 99%, respectively (11). However, this method is traumatic and requires high skills to performers. Moreover, ^18^F-FDG PET/CT had a satisfying diagnostic value of ILN metastasis, with a sensitivity and specificity of 88%–100% and 98%–100%, respectively (12, 13). However, PET/CT is expensive and difficult to promote in primary hospitals. For non-palpable ILNs, sentinel lymph node biopsy guided by SPECT/CT, in which radionuclide agents were injected into glans to locate sentinel lymph nodes, was available clinically, with a sensitivity value of 88.8% and a specificity value of 86.7% on metastasis diagnosis (14, 15).

Previous literature reported that conventional CT had no value for ILN metastatic diagnosis (10). It was used only in the situation that the physical examination or ultrasonography (US) might be unreliable, such as patients with obesity or patients who had prior inguinal surgery (16). However, in clinical practice, we found that positive ILN generally had higher CT HU than negative ones. Therefore, the purpose of this study was to investigate the application of CT HU on ILN metastasis diagnosis in penile cancer.

We matched biopsied lymph nodes with pelvic CT images. As palpable ILNs were located in the superior and central inguinal zones, with most in the medial superior zone (17), they can be easily identified in CT transverse section. According to the adjacent relationship between lymph nodes and fix anatomies, such as femoral artery, pubic symphysis, sartorius muscle, and spermatic cord, target lymph nodes were precisely located on CT images.

To measure target ILN CT HU, diameter, and area, we manually outlined the edges by hand in Neusoft PACS software. The outline was sharp and clear because ILNs were imbedded in fat tissue, which had much lower density than lymph nodes in CT. In this study, we found metastatic ILNs had larger average and maximum CT HU in both NC-CT and ACE-CT, with statistically significant differences. The hypothetic reason is that normal lymph nodes contain more adipose tissue, whereas metastatic ones are substantial tumors, and their blood supply is much richer than negative ones. However, average CT HU would change according to different outline traces by different doctors. In contrast, maximum CT HU stayed relatively constant. In this study, maximum NC-CT HU (cutoff: 51.5) had the highest sensitivity as 0.897 among the six parameters. The result was very close to the method of fine-needle aspiration cytology guided by ultrasonography (10). Notably, average ACE-CT HU was excellent in both sensitivity and specificity, therefore possessing the highest accuracy as 0.857, which meant that average ACE-CT HU would be a potentially excellent diagnostic indicator. In brief, CT HU was proved as a simple and favorable diagnostic method for ILN metastasis.

The size of lymph node is also an important index to evaluate metastasis, and the parameter of short axis diameter is commonly used in clinical work (18, 19). We found that the diameter and area of positive ILNs were larger than negative ones (P < 0.001), which was consistent with one previous report (20). In this study, the specificity of diameter and area was high (0.956), but the sensitivity (0.615 and 0.564) was lower than CT HU parameters. Our results showed that 26.4% (14/53) of lymph nodes with diameter less than 15 mm and 28.3% (17/60) with area less than 150 mm^2^ were metastatic, which means that many positive ILNs would be missed if judged only by diameter or area. A previous research also displayed that 20% of the non-palpable lymph nodes were found to be metastasis (6).

Another finding displayed in this study was that primary tumor differentiation was significantly related to ILN metastasis. Previous researches have proven this conclusion (21, 22). Eleven of the 12 patients with poorly differentiated carcinoma had metastasis, regardless of T stage. Because this study only included T1 and T2 patients, it could not compare the weight of stage and tumor differentiation to ILN metastasis. However, it still prompted that a high risk of ILN metastasis existed in patients with low differentiated primary tumor.

This study has several limitations. Firstly, there were only a few subjects. Because penile cancer is a rare disease, the sample size was small. The accuracy of the results may not be sufficient. Secondly, HU values depend on the CT machine, imaging conditions, and image processing software, which may differ among institutions. The definition of contrast-enhanced arterial-phase varies as well. Different scanning time after the injection of contrast agents could cause different CT HUs. Thirdly, we only explored CT HU of palpable ILNs in the study. As non-palpable lymph nodes cannot be precisely matched with CT images, therefore, whether CT HU has the same diagnostic value in all ILNs needs further exploration.

## 5 Conclusion

CT HU is valuable for the diagnosis of palpable ILN metastasis in patients with newly diagnosed penile cancer. We found average ACE-CT HU has the highest accuracy, and maximum NC-CT HU has the highest sensitivity. These two parameters would provide a convenient application in clinical practice.

## Data Availability

The original contributions presented in the study are included in the article/**Supplementary Material**. Further inquiries can be directed to the corresponding author.

## Supplementary material

Supplementary Figure 1 The frozen section pathology image of metastatic inguinal lymph node in penile cancer patients.
