# Supplementary figures and images for: The clinical value of computed tomography Hounsfield unit for diagnosing palpable inguinal lymph node metastasis in patients with penile cancer
Source: Front Oncol. 2025 Feb 10;15:1388390. doi: 10.3389/fonc.2025.1388390 (PMC11847666; doi:10.3389/fonc.2025.1388390)

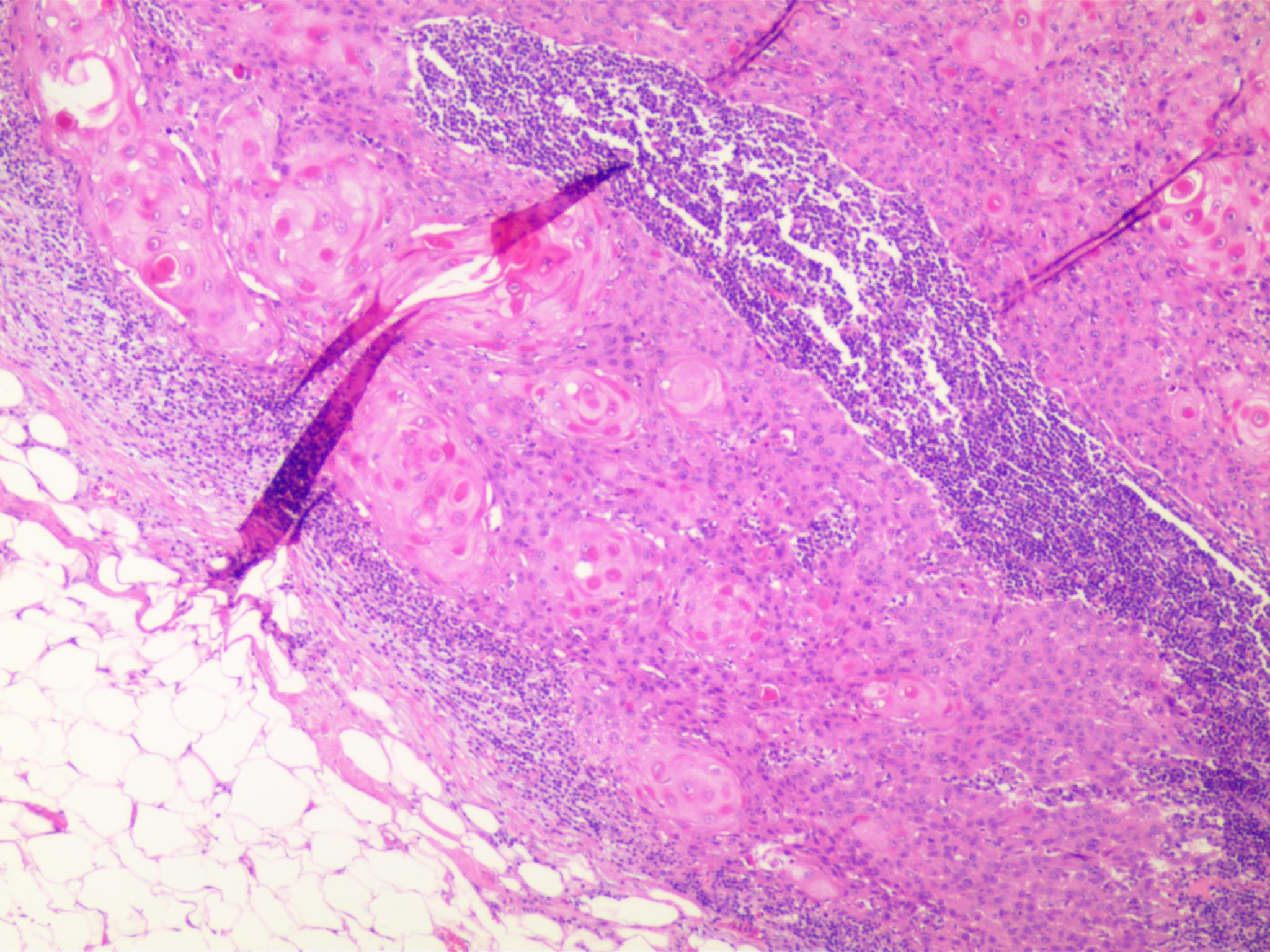

Supplement: Supplementary Figure 1 — The frozen section pathology image of metastatic inguinal lymph node in penile cancer patients. [file Image1.jpeg]
